# Analysis of Drug-Resistant Bacteria Seasonality in Japan Using Financial Time Series Analysis Method: A Nationwide Longitudinal Study

**DOI:** 10.1155/cjid/5590467

**Published:** 2025-02-28

**Authors:** Hiroshi Ito, Jura Oshida, Minori Fujita, Daiki Kobayashi

**Affiliations:** Department of Internal Medicine, Tokyo Medical University Ibaraki Medical Center, Inashiki District, Ibaraki Prefecture, Ami, Japan

**Keywords:** drug-resistant bacteria, Japan, seasonality

## Abstract

**Introduction:** Bacterial infections exhibit seasonal variation, particularly in respiratory pathogens; however, whether similar trends exist for bacterial infections and resistance in Japan is unclear. This study examined seasonal and annual patterns of bacterial isolation rates and antimicrobial resistance in Japanese hospitals, utilizing data from the Ministry of Health, Labour, and Welfare's Nosocomial Infection Control Surveillance Project (JANIS) between 2014 and 2020.

**Methods:** Data from JANIS included isolation rates and antimicrobial resistance for four bacterial species: *Staphylococcus aureus*, *Escherichia coli*, *Klebsiella pneumoniae*, and *Pseudomonas aeruginosa*. We modeled seasonal and annual trends using a generalized autoregressive conditional heteroskedasticity (GARCH) (1, 1) model, controlling for hospital size. Analyses examined seasonal and annual trends in isolation rates and resistance patterns, including methicillin-resistant *S. aureus* (MRSA), multidrug-resistant *P. aeruginosa* (MDRP), and carbapenem-resistant *P. aeruginosa* (CRPA), among others.

**Results:** The isolation rate of *S. aureus* decreased annually, with the most pronounced decline observed from the second to the fourth quarters, particularly in smaller hospitals. The isolation rates of *E. coli* and *K. pneumoniae* increased annually, with significant seasonal peaks in the third and fourth quarters. Antimicrobial resistance showed annual declines for MRSA, MDRP, and CRPA, particularly in smaller hospitals. However, resistance rates for third-generation cephalosporin-resistant *E. coli* and *K. pneumoniae* increased during the study period.

**Conclusion:** This study demonstrates the distinct seasonal and annual trends in bacterial isolation and antimicrobial resistance in Japan. Smaller hospitals showed higher resistance rates, likely because of limited antimicrobial stewardship resources, underscoring the need for targeted interventions in these settings. These findings highlight the importance of monitoring seasonal patterns in bacterial infections and resistance to inform effective infection control and antimicrobial stewardship strategies.

## 1. Introduction

Seasonality is closely associated with infectious diseases, particularly respiratory virus infections, as documented across multiple studies. For instance, influenza A and B viruses exhibit a single annual seasonal peak during the winter months in both hemispheres. In the Northern hemisphere, influenza peaks from December to March, while in the Southern hemisphere, it peaks from June to August [1, 2]. Furthermore, common cold coronaviruses predominantly circulate during winter and spring in temperate climates, with low-level circulation throughout the year [3]. While circulating throughout the year, rhinoviruses and adenoviruses often peak in autumn and winter in temperate climates.

Seasonality has also been reported in bacterial infections, especially those caused by *Staphylococcus aureus* [4, 5]. Notably, seasonality has been reported in soft tissue infections caused by *S. aureus*. For example, a single-center observational study of patients with *S. aureus* soft tissue infections in Greece and South India found a peak in summer [6, 7]. Conversely, a single-center observational study in Norway showed peak occurrence in autumn [8], suggesting that the seasonality of *S. aureus* soft tissue infections may vary by country.

In addition to *S. aureus* infections, seasonality has been reported in Gram-negative bacilli infections [9], and a multicenter observational study in the United States showed an association between summer months and high temperatures with increased Gram-negative bacilli bacteremia [10]. Furthermore, a retrospective observational study in Japan reported that *Clostridioides difficile* infections were more likely to occur in summer [11]. Although the reasons for this seasonality remain unclear, hypotheses include seasonal variations in antibiotic use [12] and environmental factors such as temperature and humidity [13].

Despite these findings, the seasonality of drug-resistant bacteria remains poorly understood. Limited studies suggest that resistance of *E*. *coli* to certain antibiotics increases from autumn to winter, potentially due to higher antibiotic prescriptions for respiratory infections, which elevate selective pressure on *E. coli* [14, 15]. Furthermore, many studies on the seasonality of bacterial infections have been primarily single-center or multicenter observational studies, with limited research using national data. Therefore, to address these gaps, we investigated whether there is seasonality in drug-resistant bacteria frequency in Japanese hospitals using data from Japan Nosocomial Infections Surveillance (JANIS), the Ministry of Health, Labour, and Welfare.

## 2. Methods

We conducted a nationwide longitudinal study using data from JANIS (https://janis.mhlw.go.jp/index.asp) to examine whether there is seasonality in drug-resistant bacteria frequency in Japanese hospitals. JANIS is a nationwide survey conducted by the Ministry of Health, Labor, and Welfare to comprehend the overall epidemiology of drug-resistant bacteria in Japan [16, 17]. As of January 2024, over 3207 hospitals were participating, covering approximately 40% of all hospitals in Japan. Data from bacterial drug susceptibility tests conducted at each healthcare facility were aggregated by the National Institute of Infectious Diseases. These results are released annually in a public report, with feedback reports provided to participating facilities [18]. Since the 2015 revision of Japan's medical fee schedule, participation in JANIS has been mandatory for facilities that meet infection prevention criteria, resulting in a yearly increase in participating healthcare facilities. In accordance with publicly available data usage policies, JANIS data can be utilized for publication purposes, without requiring explicit consent or permission from the Ministry of Health, Labor, and Welfare. Furthermore, open data analysis in Japan does not require ethics committee approval, which was thus waived for this study.

Initially, we extracted the number of isolates and the proportion of total specimens submitted for *S. aureus*, *Escherichia coli*, *Klebsiella pneumoniae*, and *Pseudomonas aeruginosa* in registered hospitals in Japan from January 2014 to December 2020 using JANIS data. Duplicate cases were excluded when data were made publicly available in JANIS. Additionally, we extracted the proportions of methicillin-resistant *S. aureus* (MRSA) isolates to the total number of *S*. *aureus* isolates, the proportion of third-generation cephalosporin- and fluoroquinolone-resistant strains to the number of *E*. *coli* isolates, the proportion of third-generation cephalosporin-resistant strains to the number of *K*. *pneumoniae* isolates, and the proportion of multidrug- and carbapenem-resistant strains to the number of *P*. *aeruginosa* isolates. However, we did not focus on vancomycin-resistant *S. aureus*, vancomycin-resistant enterococci, or carbapenem-resistant *Enterobacteriaceae*, which are rare in Japan.

In accordance with JANIS aggregation methods, isolation rates were defined as the proportion of patients with the target bacteria among all patients who submitted samples each quarter. Antimicrobial resistance (AMR) rates were defined as the number of patients from whom drug-resistant bacteria were isolated as a proportion of the total number of patients with the target bacteria, based on the breakpoints in the Clinical and Laboratory Standards Institute M100-S22 [19]. If multiple bacteria were isolated from the same sample, each bacterial type was counted separately as an individual instance of isolation.

To model the seasonality and variability in the isolation rate of drug-resistant bacteria, we utilized a generalized autoregressive conditional heteroskedasticity (GARCH) (1, 1) model, a specific type of GARCH model that captures time-dependent volatility and fluctuations in longitudinal data [20]. This approach is particularly suitable for epidemiological studies where changes in bacterial isolation rates may vary significantly over time. GARCH accounts for both long-term trends and seasonal variations in resistance rates, enhancing analytical precision and robustness. We also included hospital size in the models, specifically whether hospitals had > or < 200 beds, to capture the impact of facility scale on resistance rates. The analysis was conducted using R (The R Foundation for Statistical Computing, Vienna, Austria, version 4.4.1). Detailed R codes used in this analysis are listed in Supporting Table 1.

## 3. Results

Between 2014 and 2020, the number of healthcare facilities participating in JANIS increased steadily from 883 to 2,167, accompanied by an increase in the total number of specimens submitted (Table 1). Blood samples consistently constituted the largest category, followed by the respiratory, urine, stool, and cerebrospinal fluid samples. The number of positive results for bacterial detection has grown from approximately 2.1 million in 2014 to over three million by 2020.

### 3.1. Bacterial Isolation Rates

**Figure 1:**
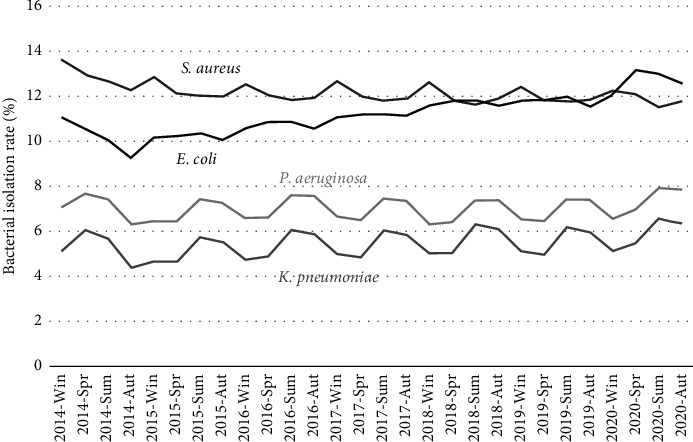
Bacterial isolation rates.

The GARCH (1,1) analysis of bacterial isolation rates revealed significant trends across several bacterial species (Figure 1; Table 2). *S*. *aureus* exhibited a negative annual trend (coefficient, −0.00057; *p*=0.047) and notable decreases in isolation rates during the second (coefficient, −0.0075; *p* < 0.001), third (coefficient, −0.012; *p* < 0.001), and fourth quarters (coefficient, −0.0093; *p* < 0.001), with hospitals having < 200 beds showing higher rates (coefficient, 0.039; *p* < 0.001). In contrast, *E*. *coli* demonstrated a positive annual trend (coefficient, 0.0063; *p* < 0.001), although seasonal variations were not significant; hospitals with < 200 beds had elevated rates (coefficient, 0.036; *p* < 0.001). For *K*. *pneumoniae*, a positive annual trend was also observed (coefficient, 0.0014; *p* < 0.001), with significant increases in the third (coefficient, 0.013; *p* < 0.001) and fourth quarters (coefficient, 0.0098; *p* < 0.001) and higher rates in hospitals with < 200 beds (coefficient, 0.016, *p* < 0.001). Lastly, *P*. *aeruginosa* did not show a significant annual trend (coefficient, 0.00015; *p*=0.56); however, significant increases occurred in the third (coefficient, 0.011; *p* < 0.001) and fourth quarters (coefficient, 0.0094; *p* < 0.001), with higher rates in hospitals with < 200 beds (coefficient, 0.021; *p* < 0.001).

### 3.2. AMR Rates

**Figure 2:**
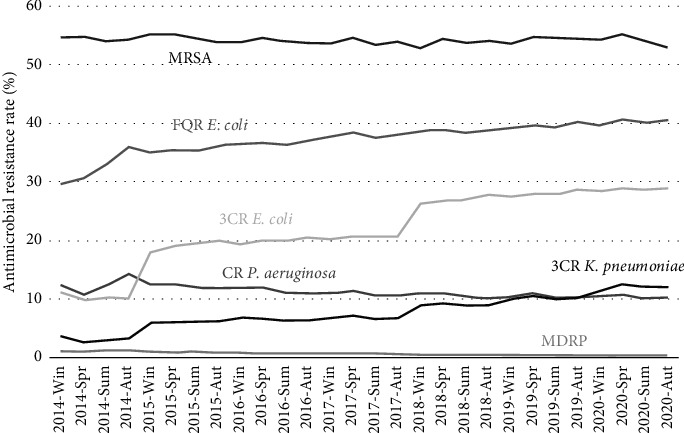
Antimicrobial resistance rates. 3CREC, third-generation cephalosporin-resistant *E. coli*; 3CRKP, third-generation cephalosporin-resistant *K. pneumoniae*; CRPA, carbapenem-resistant *P. aeruginosa*; FQREC, fluoroquinolone-resistant *E. coli*; MDRP, multidrug-resistant *P. aeruginosa*; MRSA, methicillin-resistant *S. aureus*.

The GARCH (1, 1) analysis of AMR rates identified significant trends in various bacterial strains influenced by year, season, and hospital size (Figure 2; Table 3). For MRSA, resistance rates slightly decreased over time (coefficient, −0.0020; *p*=0.017), with a seasonal increase from April to June (coefficient, 0.013; *p*=0.0081), and significantly higher resistance rates in hospitals with < 200 beds (coefficient, 0.096; *p* < 0.001). Multidrug-resistant *P*. *aeruginosa* (MDRP) also displayed a year-on-year decline (coefficient, −0.0012; *p* < 0.001), with a notable drop in from October to December (coefficient, −0.0010; *p*=0.0064) and slightly lower rates in smaller hospitals (coefficient, −0.00071; *p*=0.0089). Carbapenem-resistant *P. aeruginosa* (CRPA) resistance rates significantly decreased over time (coefficient, −0.0047; *p* < 0.001), with hospitals having < 200 beds again showing higher resistance (coefficient, 0.011; *p* < 0.001). Third-generation cephalosporin-resistant *K*. *pneumoniae* (3CRKP) resistance rates increased over the years (coefficient, 0.015; *p* < 0.001), with smaller hospitals displaying higher rates of resistance (coefficient, 0.020; *p* < 0.001). Third-generation cephalosporin-resistant *E*. *coli* (3CREC) also saw a strong upward trend over time (coefficient, 0.031; *p* < 0.001), with smaller hospitals showing elevated resistance rates (coefficient, 0.041; *p* < 0.001). Lastly, fluoroquinolone-resistant *E. coli* (FQREC) exhibited a positive annual trend (coefficient, 0.0084; *p* < 0.001), with a seasonal increase from October to December (coefficient, 0.0088; *p*=0.072) and higher resistance in smaller hospitals (coefficient, 0.095; *p* < 0.001).

## 4. Discussions

We identified significant annual and seasonal trends in bacterial isolation and AMR rates in Japan. *S. aureus* isolation declined annually with seasonal dips, whereas *E. coli* and *K. pneumoniae* increased over time. *P. aeruginosa* showed late-year peaks. Regarding resistance, MRSA, MDRP, and CRPA decreased annually but exhibited seasonal variation and were generally more prevalent in smaller hospitals. Resistance to third-generation cephalosporins increased in *K. pneumoniae* and *E. coli*, while fluoroquinolone resistance rose in *E. coli*. Smaller hospitals exhibited notably higher resistance rates.

*S. aureus* isolation rates decreased annually, whereas *E. coli* and *K. pneumoniae* increased over time. The variation in isolation rates suggests that the effectiveness of infection prevention measures, such as adherence to standard precautions, differs by bacterial species. Additionally, Japan's aging population may also contribute differently to these trends, with some infections decreasing due to enhanced infection prevention practices and others increasing due to age-related susceptibility. Since the 1980s, Japan, has strengthened its infection control system in response to the rising MRSA infection rates [21]. In the 2010s, national infection control measures became more widespread, with related practices incentivized through medical reimbursements. These measures were likely effective against *S. aureus*, which easily infects the skin and mucous membranes, but may have been less effective for *E. coli* and *K. pneumoniae*, which originate in the intestinal tract. Meanwhile, Japan's aging population, with 28% of individuals aged ≥ 65 years in 2017 and expected to reach 40% by 2060 [22, 23], may contribute to increasing vulnerability to infections, especially *E. coli* and *K. pneumoniae*. *S. aureus* infections may decrease because the preventive effects of infection control measures outweigh the increased infection risk associated with an aging population.

Regarding seasonality, *S. aureus* exhibited higher isolation rates in winter, whereas *K. pneumoniae* and *P. aeruginosa* were more prevalent in the summer and fall. Winter in Japan is associated with increased cases of myocardial infarction and severe strokes, often requiring intensive care and medical device use, potentially contributing to higher *S. aureus* infection rates [24, 25]. Conversely, Japan's warm and humid summer-to-fall conditions, with average temperatures around 30°C and humidity levels of 70%–80% [26], may favor the growth of Gram-negative bacteria like *K. pneumoniae* and *P. aeruginosa* [27]. Previous studies have also reported seasonal increases in Gram-negative bacteria, particularly in respiratory and urinary specimens during summer in Japan [28].

There was a yearly decrease in AMR, MRSA, MDRP, and CRPA, whereas 3CRKP, 3CREC, and FQREC showed an upward trend. The decline in certain resistant bacteria could be due to antimicrobial stewardship programs, including the restriction of specific antibiotics, such as carbapenems and anti-MRSA drugs, which may have reduced the selective pressure for resistance in these bacterial strains [29, 30]. However, resistance may have risen in bacterial strains unaffected by such restrictions, possibly due to increased usage of substitute antibiotics. Additionally, MRSA was more common in the spring, possibly linked to seasonal staff turnover, which could temporarily affect infection control proficiency. MDRP increased during winter, potentially due to a rise in severe cases and greater use of medical devices in intensive care, as noted earlier.

From a time-series perspective, our findings indicate that bacterial isolation and AMR rates fluctuate in a manner similar to financial market trends. By applying financial time series models such as GARCH (1, 1), we quantified fluctuations in detection rates and assess how shocks to resistance trends persist over time. The presence of conditional heteroskedasticity in AMR trends suggests that past variations in resistance influence future fluctuations, highlighting the importance of dynamic monitoring. Notably, resistance to third-generation cephalosporins and fluoroquinolones exhibited increasing trends with sustained variability, suggesting persistent selective pressures in certain settings. These findings underscore the potential for advanced time series modeling in AMR surveillance, allowing for more precise risk assessment and forecasting of resistance trends.

Notably, hospitals with < 200 beds had higher rates of resistant bacteria than those with > 200 beds, with the exception of MDRP. This could stem from challenges in implementing antimicrobial stewardship programs owing to the absence of infectious disease specialists. As of 2024, Japan has fewer than 2000 infectious disease specialists [31], with approximately one-third working in university hospitals, underscoring both their scarcity and uneven distribution [32]. For MDRP, however, larger hospitals may see higher rates because of the more frequent use of intensive care treatments.

Our study has some limitations. First, publicly available JANIS data restricted our ability to include certain variables. For example, data for 2021 and 2022 were only available as annual reports, excluding quarterly details. Nevertheless, the 7 years of data analyzed (2014–2020) provided a robust sample size for time-series analysis. Second, we could not stratify the data by sample type, as JANIS does not publish AMR data by specimen type. Therefore, our analysis likely included data on colonized bacteria in the sputum or asymptomatic bacteriuria, which should be considered when interpreting our findings. Finally, MDRP and other low-frequency pathogens may exhibit trends that lack clinical significance, emphasizing the importance of continuous AMR surveillance.

In conclusion, we described annual and seasonal trends in isolation rates of bacteria and antimicrobial-resistant bacteria from cultured specimens. We found that *S. aureus* was more prevalent in winter, while *K. pneumoniae* and *P. aeruginosa* were more common in summer and fall. However, AMR exhibited no distinct seasonal patterns, except for MRSA and MDRP. Among Enterobacteriaceae, resistance rates to third-generation cephalosporins and fluoroquinolones have increased annually, indicating potential opportunities to implement antimicrobial stewardship interventions.

## Figures and Tables

**Table 1:** Backgrounds of JANIS annual data.

	2014	2015	2016	2017	2018	2019	2020
Facilities, *n*	883	1435	1643	1795	1947	2075	2167
Specimens, *n*	5,087,263	7,286,127	7,857,602	8,013,062	8,222,470	8,416,486	7,959,180
Blood, *n*	1,549,836	2,311,915	2,560,343	2,689,342	2,832,767	2,938,267	2,855,668
Respiratory, *n*	1,472,016	2,092,027	2,220,093	2,214,026	2,221,362	2,231,419	1,944,725
Urine, *n*	613,457	902,325	1,003,895	1,043,587	1,099,593	1,145,879	1,155,931
Stool, *n*	398,950	548,310	581,065	569,625	575,737	597,171	537,189
Cerebrospinal fluid, *n*	62,796	83,604	91,144	86,857	86,877	85,785	80,188
Others, *n*	990,208	1,347,946	1,401,062	1,409,625	1,406,134	1,417,965	1,385,479
Positive results, *n*	2,104,250	2,980,643	3,182,773	3,211,305	3,257,488	3,281,861	3,077,464
Isolated bacteria, *n*	3,871,194	5,412,369	5,717,611	5,729,996	5,787,149	5,815,803	5,427,892

Abbreviation: JANIS, Japan Nosocomial Infections Surveillance.

**Table 2:** GARCH analysis of bacterial isolation rates.

Bacteria	Variables	Coefficients	95% CIs		*p* value
*S. aureus*	**Year**	**−0.00057**	**−0.0011**	**−0.000019**	**0.047**
	Jan-Mar	0.00			
	**Apr-Jun**	**−0.0075**	**−0.011**	**−0.0044**	**< 0.001**
	**Jul-Sep**	**−0.012**	**−0.015**	**−0.0087**	**< 0.001**
	**Oct-Dec**	**−0.0093**	**−0.012**	**−0.0062**	**< 0.001**
	≥ 200 beds	0.00			
	**< 200 beds**	**0.039**	**0.037**	**0.041**	**< 0.001**

*E. coli*	**Year**	**0.0063**	**0.0053**	**0.0074**	**< 0.001**
	Jan-Mar	0.00			
	Apr-Jun	0.0030	−0.0030	0.0089	0.33
	Jul-Sep	0.0035	−0.0024	0.0094	0.25
	Oct-Dec	0.00032	−0.0056	0.0062	0.92
	≥ 200 beds	0.00			
	**< 200 beds**	**0.036**	**0.031**	**0.040**	**< 0.001**

*K. pneumoniae*	**Year**	**0.0014**	**0.00089**	**0.0019**	**< 0.001**
	Jan-Mar	0.00			
	Apr-Jun	0.0017	−0.0011	0.0044	0.24
	**Jul-Sep**	**0.013**	**0.0010**	**0.015**	**< 0.001**
	**Oct-Dec**	**0.0098**	**0.0070**	**0.012**	**< 0.001**
	≥ 200 beds	0.00			
	**< 200 beds**	**0.016**	**0.014**	**0.018**	**< 0.001**

*P. aeruginosa*	Year	0.00015	−0.00035	0.00066	0.56
	Jan-Mar	0.00			
	Apr-Jun	0.0018	−0.0011	0.0046	0.235
	**Jul-Sep**	**0.011**	**0.0081**	**0.014**	**< 0.001**
	**Oct-Dec**	**0.0094**	**0.0065**	**0.012**	**< 0.001**
	≥ 200 beds	0.00			
	**< 200 beds**	**0.021**	**0.018**	**0.023**	**< 0.001**

*Note:* Bold values denote statistical significance at the *p* < 0.05 level.

Abbreviations: CI, confidence interval; GARCH, generalized autoregressive conditional heteroskedasticity.

**Table 3:** GARCH analysis of antimicrobial resistance rates.

Bacteria	Variables	Coefficients	95% CIs		*p* value
MRSA	**Year**	**−0.0020**	**−0.0036**	**−0.00042**	**0.017**
	Jan-Mar	0.00			
	**Apr-Jun**	**0.013**	**0.0037**	**0.022**	**0.0081**
	Jul-Sep	0.0040	−0.0050	0.013	0.39
	Oct-Dec	0.00098	−0.0081	0.010	0.83
	≥ 200 beds	0.00			
	**< 200 beds**	**0.096**	**0.090**	**0.10**	**< 0.001**

MDRP	**Year**	**−0.0012**	**−0.0013**	**−0.0011**	**< 0.001**
	Jan-Mar	0.00			
	Apr-Jun	−0.00043	−0.0012	0.00029	0.24
	Jul-Sep	−0.00029	−0.0010	0.00043	0.43
	**Oct-Dec**	**−0.0010**	**−0.0018**	**−0.00033**	**0.0064**
	≥ 200 beds	0.00			
	**< 200 beds**	**−0.00071**	**−0.0012**	**−0.00020**	**0.0089**

CRPA	**Year**	**−0.0047**	**−0.0057**	**−0.0036**	**< 0.001**
	Jan-Mar	0.00			
	Apr-Jun	0.00047	−0.0054	0.0063	0.88
	Jul-Sep	−0.0060	−0.012	−7.7 × 10^−5^	0.053
	Oct-Dec	−0.0051	−0.011	0.00079	0.096
	≥ 200 beds	0.00			
	**< 200 beds**	**0.011**	**0.0064**	**0.015**	**< 0.001**

3CRKP	**Year**	**0.015**	**0.013**	**0.017**	**< 0.001**
	Jan-Mar	0.00			
	Apr-Jun	0.0032	−0.0067	0.013	0.53
	Jul-Sep	−0.0027	−0.013	0.0072	0.60
	Oct-Dec	−0.0012	−0.011	0.0086	0.81
	≥ 200 beds	0.00			
	**< 200 beds**	**0.020**	**0.013**	**0.027**	**< 0.001**

3CREC	**Year**	**0.031**	**0.027**	**0.035**	**< 0.001**
	Jan-Mar	0.00			
	Apr-Jun	0.0028	−0.019	0.024	0.80
	Jul-Sep	0.0019	−0.020	0.023	0.86
	Oct-Dec	0.0065	−0.015	0.028	0.56
	≥ 200 beds	0.00			
	**< 200 beds**	**0.041**	**0.026**	**0.056**	**< 0.001**

FQREC	**Year**	**0.0084**	**0.0067**	**0.010**	**< 0.001**
	Jan-Mar	0.00			
	Apr-Jun	0.0065	−0.0029	0.016	0.18
	Jul-Sep	−0.00056	−0.0099	0.0088	0.91
	Oct-Dec	0.0088	−0.00060	0.018	0.072
	≥ 200 beds	0.00			
	**< 200 beds**	**0.095**	**0.088**	**0.10**	**< 0.001**

*Note:* Bold values denote statistical significance at the *p* < 0.05 level.

Abbreviations: 3CREC, third-generation cephalosporin-resistant *E. coli*; 3CRKP, third-generation cephalosporin-resistant *K. pneumoniae*; CI, confidence interval; CRPA, carbapenem-resistant *P. aeruginosa*; FQREC, fluoroquinolone-resistant *E. coli*; GARCH, generalized autoregressive conditional heteroskedasticity; MDRP, multidrug-resistant *P. aeruginosa*; MRSA, methicillin-resistant *S. aureus*.

## Data Availability

The data that support the findings of this study are available in Japan Nosocomial Infections Surveillance at https://janis.mhlw.go.jp/index.asp. These data were derived from the following resources available in the public domain: Public information by number of beds, https://janis.mhlw.go.jp/report/kensa.html.

## Supporting Information

Supporting Table 1: Detailed R code used in the GARCH analysis.
