# Supplementary material for: Analysis of Drug-Resistant Bacteria Seasonality in Japan Using Financial Time Series Analysis Method: A Nationwide Longitudinal Study
Source: Can J Infect Dis Med Microbiol. 2025 Feb 28;2025:5590467. doi: 10.1155/cjid/5590467 (PMC11986954; doi:10.1155/cjid/5590467)
Supplement: Supporting Information — Additional supporting information can be found online in the Supporting Information section. [file 5590467.f1.docx]

Supplementary Table 1. Detailed R code used in the GARCH analysis

library(rugarch)

library(broom)

library(dplyr)

data <- read.csv("Data.csv")

# Function to fit a linear model and then apply GARCH(1,1) to residuals

fit_garch_with_regression <- function(df, bacteria) {

# Step 1: Linear Regression

lm_model <- lm(as.formula(paste(bacteria, "~ Year + Season + Hospital")), data = df)

lm_summary <- summary(lm_model)

# Extract coefficients and other statistics from linear model

lm_results <- tidy(lm_summary)

# Step 2: GARCH(1,1) on residuals

residuals <- lm_model$residuals

spec <- ugarchspec(

variance.model = list(model = "sGARCH", garchOrder = c(1, 1)),

mean.model = list(armaOrder = c(0, 0), include.mean = TRUE),

distribution.model = "norm"

)

fit <- ugarchfit(spec, data = residuals)

# Extracting the GARCH coefficients, standard errors, etc.

garch_coef <- coef(fit)

garch_se <- sqrt(diag(vcov(fit)))

garch_z_value <- garch_coef / garch_se

garch_p_value <- 2 * pnorm(-abs(garch_z_value))

garch_ci_lower <- garch_coef - qnorm(0.975) * garch_se

garch_ci_upper <- garch_coef + qnorm(0.975) * garch_se

garch_results <- data.frame(

term = names(garch_coef),

estimate = garch_coef,

std.error = garch_se,

statistic = garch_z_value,

p.value = garch_p_value,

conf.low = garch_ci_lower,

conf.high = garch_ci_upper

)

# Combine the linear model results and GARCH results

combined_results <- bind_rows(lm_results, garch_results)

return(combined_results)

}

# Prepare the data for each bacteria and fit the model

results <- list()

bacteria_cols <- c("SA", "PA", "KP", "EC", "MRSA", "MDRP", "CaRPA", "CeRKP", "CeREC", "FQREC")

for (bacteria in bacteria_cols) {

df <- data %>%

select(Year, Season, Hospital, all_of(bacteria))

# Fit the model and store results

fit_result <- fit_garch_with_regression(df, bacteria)

results[[bacteria]] <- fit_result

}

# Combine results into a data frame

final_results <- bind_rows(results, .id = "Bacteria")

# Output the results

print(final_results, n = Inf)
